# Biofluid Biomarkers in the Prognosis of Chronic Subdural Hematoma: A Systematic Scoping Review

**DOI:** 10.3390/diagnostics13142449

**Published:** 2023-07-22

**Authors:** Georgios Georgountzos, Ioannis Gkalonakis, Lykourgos Anastasopoulos, George Stranjalis, Theodosis Κalamatianos

**Affiliations:** 1 Department of Neurosurgery, General Hospital of Nikaia-Piraeus “Agios Panteleimon”, 18454 Athens, Greece; 2 Clinical and Experimental Neuroscience Research Group, Department of Neurosurgery, National and Kapodistrian University of Athens, Evangelismos Hospital, 10676 Athens, Greece; 3 Department of Neurosurgery, National and Kapodistrian University of Athens, Evangelismos Hospital, 10676 Athens, Greece; 4 Hellenic Centre for Neurosurgery Research, “Professor Petros S. Kokkalis”, 10675 Athens, Greece

**Keywords:** chronic subdural hematoma, prognosis, recurrence, biomarker

## Abstract

The present systematic scoping review aimed at mapping and analyzing the available literature on biological fluid (biofluid) biomarkers showing promise in the prediction of chronic subdural hematoma (cSDH) recurrence and the prognosis of neurological/functional patient outcome. Twenty-three studies published between 2003 and 2023 investigating a diverse range of biomarkers in hematoma fluid and/or the circulation in 3749 patients were included. Immune cell populations and inflammatory/anti-inflammatory cytokines comprised the most studied category of biomarkers displaying significant findings. A notable time trend in biomarker studies was a recent shift in research focus towards the analysis of circulating biomarkers. Several biomarkers were indicated as independent predictors of cSDH recurrence and/or functional/neurological outcome, including circulating fibrinogen degradation products (FDP), brain natriuretic peptide (BNP-1) and high-density lipoprotein (HDL), as well as blood urea nitrogen (BUN) and the ratios of blood neutrophil to lymphocyte (NLR) or red blood cell distribution width to platelet count (RPR). While studies on cSDH prognostic biomarkers have gained, in recent years, momentum, additional multicenter prospective studies are warranted to confirm and extend their findings. The identification of prognostic biofluid biomarkers in cSDH is an active field of research that may provide future tools, guiding clinical decisions and allowing for the design of treatments based on risk stratification.

## 1. Introduction

Chronic subdural hematoma (cSDH) is a common neurosurgical entity that typically affects elderly patients and is increasing in incidence. The presenting symptoms of cSDH are variable but commonly include gait disturbances and mental deterioration as well as limb weakness [1]. The diagnosis of cSDH is based on imaging indicating the presence of a hematoma in the subdural space, often in association with a history of mild or moderate head injury, weeks to months prior to hospitalization [2]. Histologically, cSDH is characterized by an external and an internal membrane, encapsulating the hematoma, both containing fibroblast layers with only the former showing the persistent presence of immature, leaky vessels that have been postulated to drive hematoma expansion [3].

Surgical evacuation remains the standard of care for cSDH with immediate results postoperatively [2]. Nevertheless, the postoperative recurrence of cSDH is not uncommon, ranging between 5 and over 30% in previous studies [2]. cSDH recurrence thus currently represents a significant nosological consideration, the more so since reoperations increase healthcare-associated costs and are a substantial burden on patient morbidity and mortality [1,4]. In this context, previous studies have sought to establish the factors associated with an increased risk of cSDH recurrence and/or which can aid in the prediction of neurological/functional outcomes. Thus far, several clinical factors, e.g., increased patient age, radiological characteristics, e.g., bilateral hematoma, and specific external membrane features, e.g., enhanced presence of angiogenic factors, have been previously indicated as risk factors for cSDH recurrence [5,6,7,8]. Additional research has focused on identifying prognostic molecular or cellular factors within the hematoma fluid and/or peripheral blood of cSDH patients. This class of potential prognostic biofluid biomarkers includes molecules and/or cellular populations that are implicated in cSDH pathogenetic and pathophysiological processes such as cerebrospinal fluid leaks, inflammation and fibrinolysis [3,9,10]. While at present no such biomarker has been validated for clinical use, the overarching goal of this research field is to provide valuable tools guiding future clinical decision making in cSDH [11].

To the best of our knowledge, this is the first systematic scoping review that maps the pertinent literature on biofluid biomarkers showing promise in postoperative cSDH prognosis. We included studies reporting differences in biomarker levels between recur-rent and non-recurrent patients as well as those indicating associations between levels and patient outcomes.

## 2. Materials and Methods

The PRISMA checklist (http://prisma-statement.org, accessed on 6 April 2023) for scoping reviews was used to address patients with chronic subdural hematoma (Participants) in terms of biomarkers measured in peripheral blood or the hematoma fluid or both (Intervention) in studies that may additionally recruit neurologically intact patients (Controls), with the aim to determine their association with disease prognosis (Outcome).

A literature search of the MEDLINE database was conducted on 4 April 2023. The search algorithm included keywords such as “chronic subdural”, “recurrence”, “prognosis” and “markers” and can be found in the Appendix A. The search did not have a language restriction and the search algorithm included free text and Medical Subject Headings (MeSH) terms. Two independent reviewers (I.G. and L.A.) screened the results of the search primarily according to the title and abstract. Any differences in opinion were resolved through discussion and by reaching a consensus with a 3rd independent reviewer (G.G.).

Inclusion criteria were set as follows: (1) study types: observational, cohort studies and randomized control studies (RCTs), (2) English-language manuscripts, (3) human studies which included surgical treatment; (4) studies that involved biofluid markers (e.g., hematoma fluid or circulation cellular/molecular markers) and (5) prognosis relevance. Exclusion criteria were (1) reviews, case reports, letters and hypotheses, (2) animal studies, (3) studies on imaging markers and (4) studies with results of no statistical significance or without statistical analysis of the results. According to these criteria, full text screening was performed by two independent reviewers (I.G. and L.A.), and when a conflict came up, a third reviewer intervened (G.G.).

Data were extracted by two independent reviewers (I.G. and L.A.) and verified by a third (G.G.) reviewer. Data extraction included DOIs, publication year, country, study type, population characteristics (sample size, age, co-morbidities), outcome measures associated with prognosis and conclusions. Reviewers aimed at incorporating studies that address the following questions and provided significant findings from different levels of analysis: (1) biomarkers the levels of which differ between patients with recurrence and those without, (2) biomarkers that have been indicated as independent risk factors for CSDH recurrence and (3) biomarkers associated with prognosis in terms of neurological/functional outcome. Studies were summarized in tables.

## 3. Results

**Figure 1:**
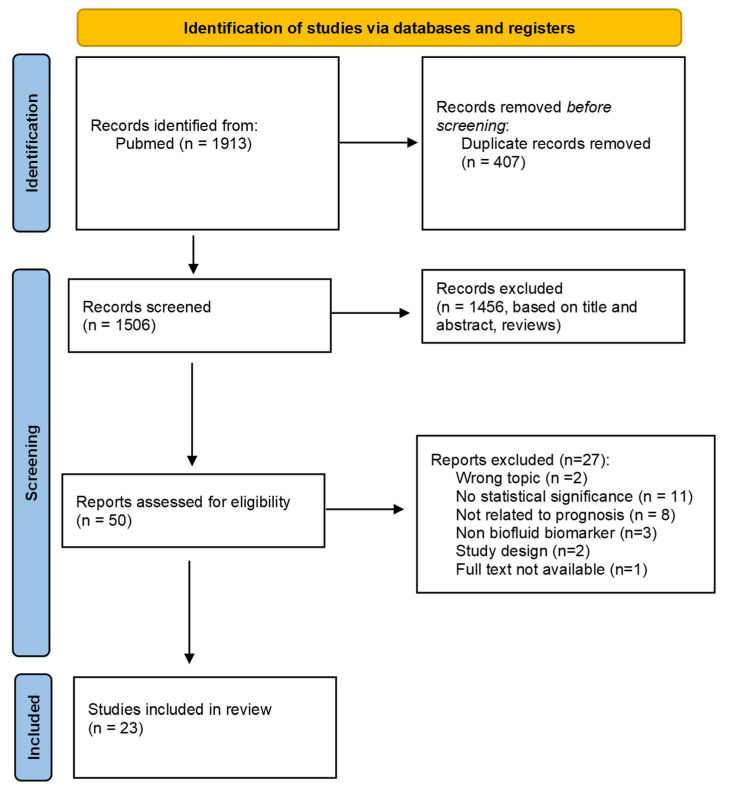
Prisma flow diagram.

A Pubmed search identified a total of 1913 articles (Figure 1). After the removal of duplicates, 1506 records remained and were screened based on title and abstract, and 50 reports were further assessed by full text reading. From these, 27 were excluded based on statistically insignificant results (*n* = 11), wrong topic (*n* = 2), not being related to prognosis (*n* = 8), non-biofluid marker (*n* = 3), study design (*n* = 2, one case report and one case series with three patients) and lack of full text availability (*n* = 1). Twenty-three studies were finally included in the review synthesis.

### 3.1. Study and Population Characteristics

Twenty-three studies on a total of 3749 patients were included in the current review (Table 1). Over half of the included studies (*n* = 12) had a prospective design, whilst no RCTs were found. The median (interquartile range, IQR) patient sample size of the included studies was 93 (60–258). The median (IQR) recurrence rate of twenty-one studies (two studies did not provide recurrence data) was 17.5% (5–21).

Studies were published in 17 different journals. The *Journal of Neurosurgery* ranked first in terms of number of published manuscripts (*n* = 5). The most common corresponding author’s address was a German one (*n* = 8).

### 3.2. Biofluids and Biomarker Types

Biomarkers were investigated in peripheral blood and/or hematoma fluid, with one study examining (additionally) hygroma fluid (Table 1).

Investigated biomarkers displaying significant results, which are summarized below, fell within one of the following categories (Table 1): (i) immune- and/or circulatory-system-associated cellular populations and/or their ratios (ten studies), (ii) fibrinolysis-/coagulation-associated molecules, complexes, degradation products or assays (six studies), (iii) cytokines (five studies) and (iv) other/miscellaneous (six studies), such as lipoproteins, ABO (blood) types, CSF markers and markers of cardiovascular/kidney/liver function.

### 3.3. Biomarker Findings

#### 3.3.1. Immune- and/or Circulatory-System-Associated Cellular Populations

Several studies reported significant findings on blood platelets (PLTs) in relation to CSDH recurrence. Thus, a prospective cohort study on 114 patients was the earliest study identified which reported significantly lower preoperative levels in patients with recurrence compared with non-recurrent cases [12]. A similar result was indicated by the univariate (but not multivariable) analysis of a more recent retrospective cohort study [13]. Two additional retrospective studies by a Japanese group incorporating larger patient cohorts indicated preoperative thrombocytopenia as an independent risk factor for CSDH recurrence [14,15].

Blood eosinophil levels were investigated by two studies. An eosinophil-rich count of ≥100 × 10^6^/L preoperatively was highlighted as an independent risk factor for cSDH recurrence in the study of Matsubara et al. [15]. In contrast, a second study on a smaller patient cohort indicated that the preoperative eosinophil count in their non-recurrent CSDH group was significantly higher than that in the recurrent group (0.14  ±  0.14 × 10^9^/L vs. 0.09  ±  0.08 × 10^9^/L) and that an eosinophil count < 150 × 10^6^/L was an independent risk factor for recurrence [16].

Three additional studies investigated peripheral blood cellular population ratios that were considered to be inflammatory biomarkers. A prospective cohort study on 61 surgically treated patients indicated that a low admission blood platelet-to-lymphocyte ratio (PLR) was associated with worse outcomes using the Glasgow outcome scale (GOS) and Lagos brain disability examination scale (LABDES) [17]. In a retrospective study on 297 patients, Guresir et al. [18] showed that circulating high red blood cell distribution width to platelet count ratio (RPR) (>0.0568) is an independent predictor of recurrence. In addition, patients with low RPR improved more in terms of Karnofsky performance status at 3 months [18]. The third study, a retrospective cohort study on 160 patients, indicated that a postoperative neutrophil-to-lymphocyte ratio (NLR) ≥ 1 (i.e., NLR that remains high compared to its preoperative value) and the postoperative absolute neutrophil counts were independently associated with recurrence [19].

Lastly, a prospective study on a small cohort (30 patients) measured preoperative and postoperative circulating endothelial progenitor cells (EPCs) and reported that patients with recurrence had a lower mean difference in postoperative and preoperative EPCs in comparison with those without relapse (MV 8.54 vs. 35.52) [20].

#### 3.3.2. Cytokines

A prospective cohort study on 66 patients indicated significantly higher levels of IL-6 in the hematoma fluid of recurrent patients [6]. Another prospective cohort study on 35 patients undergoing surgery indicated that in addition to IL-6, IL-8 hematoma levels were significantly higher in patients with recurrence [21]. Nevertheless, in the retrospective cohort of Pripp and Stanisic [10] that included 93 patients, an association between increased hematoma fluid CXCL8 (IL-8) and reduced risk of recurrence in need of reoperation was indicated. In the same study, a positive association between hematoma fluid CCL5 (also termed regulated on activation, normal T-cell expressed and secreted, RANTES) levels and recurrence as well as a negative association between IL-5, IL-13, IFN-γ and CXCL10 levels and recurrence was also indicated. In the earlier prospective cohort study of these two researchers on 57 patients, an association between increased hematoma fluid (but not serum) of a panel of biomarkers considered an anti-inflammatory index (IL-4, IL-5, IL-10, IL-13, IL-1 RA) and a reduced risk of recurrence was indicated [22]. In the retrospective cohort pilot study of Puccio et al. [11], higher IL-5 hematoma fluid levels were associated with a better outcome in terms of the extended Glasgow outcome scale (GOS-E) at 3, 6 and 12 months. Moreover, in this study, a lower concentration of hematoma RANTES (<1649.24 pg/mL) was associated with recurrence [11].

#### 3.3.3. Fibrinolysis-/Coagulation-Associated Molecules/Complexes, Products and Assays

Four prospective and two retrospective cohort studies were identified. A prospective cohort study on 60 patients investigated the levels of several biomarkers, including tissue plasminogen activator (TPA), in both hematoma fluid and peripheral blood [23]. The authors indicated that the TPA levels in the hematoma fluid of recurrent cases were significantly higher than those in non-recurrent cases [23]. A second prospective cohort study was on 18 patients with spontaneous cSDH and examined plasma F XIII activity [24]. Patients with recurrence of the spontaneous cSDH after the first operation had significantly lower FXIII activity in comparison to the non-recurrence group (47.5% vs. 78.5%, respectively). Moreover, the authors indicated a cut-off value of 68.5% in association with recurrence [24]. The third prospective study identified was on 61 patients treated surgically for cSDH [17]. This study indicated associations between elevated activated partial thromboplastin time (APTT), prothrombin time (PT) and international normalized ratio (INR) and worse outcome [17]. Reduced levels of serum fibrinogen in cases manifesting recurrence of cSDH compared with non-recurrence cases were indicated in the univariate analysis conducted by Wang et al. [25].

A retrospective cohort study on 90 patients showed that higher preoperative PT was an independent risk factor for recurrence after surgical evacuation [26]. Another retrospective cohort study on 92 patients showed that a serum fibrinogen degradation products (FDPs) value > 5 μg/mL on admission was an independent risk factor for cSDH recurrence within 90 days [27].

#### 3.3.4. Other/Miscellaneous

In the largest prospective cohort study identified (653 patients), blood urea nitrogen (BUN) levels and additional variables (PLTs, fibrinogen, leukocytes, erythrocytes, hemoglobin, creatinine) were analyzed [25]. The authors indicated that elevated postoperative BUN levels (> 6.4 mmol/L) was the only measure independently associated with cSDH recurrence [25]. In a second prospective cohort study on 119 patients, Chihi et al. showed that the preoperative plasma brain natriuretic peptide (BNP-1) level on admission is an independent predictor of poor functional outcome at the 5- to 6-month follow-up [28]. The authors of another prospective study on 75 patients undergoing burr hole hematoma evacuation for subdural hematoma or hygroma measured the levels of the CSF marker beta-trace protein (β-TP) in serum, hematoma or hygroma [9]. The authors of this study reported that when patients with hematoma and hygroma were grouped together, the β-TP levels measured during the first operation were significantly higher in recurrent cases [9].

A large retrospective cohort study on 274 patients showed that preoperative triglycerides levels and serum lipids were positively correlated with recurrence, whilst high-density lipoprotein (HDL) was negatively associated [29]. In this study, a cut-off level of HDL at 37.45 mg/dL was the only variable indicated as an independent risk factor for cSDH recurrence [29]. In the retrospective cohort study of Mainka et al. on 256 cSDH patients, dehydration status on admission (indicated by serum urea-to-creatinine ratios (U/Cr)) was highlighted as another independent predictor for recurrence [30]. Lastly, Hirai et al. retrospectively investigated the relationship between ABO type and cSDH recurrence after burr hole drainage in 320 patients. The authors showed that type A was an independent risk factor for recurrence [14].

**Table 1:** Biomarker study characteristics and findings.

Author, Year [Ref. Number]	Study Type	Sample Size, Recurrence %	Biofluids Utilized	Biomarkers Analyzed	Significant Biomarker Findings (Recurrence/Outcome)
Κönig et al., 2003 [12]	Prospective Cohort	114, 17.5%	Blood and plasma	PLTs, INR, APTT, fibrinogen, FXIII	↓ PLTs in recurrence
Frati et al., 2004 [21]	Prospective Cohort	35, 14.3%	Serum and hematoma	IL-6, IL-8	↑ Hematoma IL-6 and IL-8 in recurrence
Katano et al., 2006 [23]	Prospective Cohort	60, 8.1%	Serum and hematoma	Plasminogen, antiplasmin, TPA, PIC, HGF, VEGF, bFGF	↑ Hematoma TPA in recurrence
Kristof et al., 2008 [9]	Prospective Cohort	75, 22.7%	Serum, hematoma and hygroma	β-TP	↑ β-TP of hematoma and hygroma in recurrence
Hong et al., 2009 [6]	Prospective Cohort	66, 21.21%	Hematoma	IL-6, VEGF, bFGF	↑ IL-6 in recurrence
Song et al., 2013 [20]	Prospective Cohort	30, 20%	Blood	RBCs, WBCs, PLTs, Hb, PT, APTT, INR, TT, EPCs	↓ EPCs in recurrence
Bosche et al., 2013 [24]	Prospective Cohort	18, 33.3%	Plasma	INR, PTT, PLTs, fibrinogen, FXIII	FXIII activity 68.5% (cut-off) → recurrence (100% sensitivity, 75% specificity)
Pripp et al., 2014 [22]	Prospective Cohort	57, 12.5%	Blood and hematoma	TNF-a, IL-1b, IL-2, IL-2R, IL-6, IL-7, IL-12, IL-15, IL-17, CCL2, CXCL8, CXCL9, CXCL1 and IL-1RA, IL-4, IL-5, IL-10 and IL-13	↑ Hematoma anti-inflammatory activity (IL-4, IL-5, IL-10, IL-1 RA, IL-13) → ↓ recurrence
Pripp et al., 2017 [10]	Retrospective Cohort	93, 17.2%	Hematoma	IL-1-IL-17, TNF-a, IFN-g, CXCL8, GM-CSF, VEGF	↑ CXCL8 (IL8), CXCL10, IL-5, IL-13, IFN-γ → ↓ recurrence ↑ CCL5 (RANTES) → ↑ recurrence
Hori et al., 2018 [27]	Retrospective Cohort	92, NR	Serum	FDP, APTT, PT, D-dimer, PLTs, INR, Hb	FDP > 5 μg/mL = independent recurrence risk factor
Wang et al., 2020 [25]	Prospective Cohort	653, 14.7%	Serum	Glucose, Cr, leukocytes, neutrophils, lymphocytes, erythrocytes, Hg, PLTs, PT, INR, fibrinogen, BUN	↓ Serum leukocytes, neutrophils, PLTs, fibrinogen in recurrence BUN > 6.4 mmol/L = independent recurrence risk factor
Suero-Molina et al., 2020 [13]	Retrospective Cohort	148, 23.6%	Blood	PLTs	↓ PLTs in recurrence
Liu et al., 2021 [29]	Retrospective Cohort	274, 84.67%	Serum	TG, total cholesterol, LDL, HDL	↑ Serum TG in recurrence Low HDL = independent recurrence risk factor (HDL > 37.5 mg/dL, ↓ risk)
Martinez-Perez et al., 2021 [26]	Retrospective Cohort	90, 18.9%	Blood	PLTs, Hb, PT	↑ PT = independent recurrence risk factor
Chihi et al., 2021 [28]	Prospective Cohort	119, 13.3%	Plasma	BNP-1	BNP-1 = independent predictor for functional outcome
Hirai et al., 2021 [14]	Retrospective Cohort	307, 10.6%	Blood	PLTs, ABO type	↓ PLTs = independent recurrence risk factor; blood type A = independent recurrence risk factor (64.9% sensitivity, 58.8% specificity)
De Oliveira et al., 2022 [19]	Retrospective Cohort	160, 22.5%	Blood	Neutrophils, NLR	Absolute neutrophil count = independent recurrence risk factor; NLR ≥ 1 = independent recurrence risk factor (77.8% sensitivity, 66.7% specificity); validation on independent sample
Idowu et al., 2022 [17]	Prospective Cohort	61, NR	Blood	INR, PT, APTT, NLR, CRP, ESR, PLR	↑ APTT, ↑ PT, ↑ INR, ↓ PLR → worse outcome
Mainka et al., 2022 [30]	Retrospective Cohort	256, 32%	Serum	U/CR	U/Cr > 80 = independent recurrence risk factor
Chen et al., 2022 [16]	Retrospective Cohort	258, 14.3%	Blood	WBCs, PLTs, basophils, eosinophils	Eosinophils < 0.15 × 10^9^ = independent recurrence risk factor
Guresir et al., 2022 [18]	Retrospective Cohort	297, 12.5%	Blood	CRP, Hb, WBC, PLTs, RDW, RPR	RPR ≥ 0.0568 = independent recurrence risk factor (70.3% sensitivity, 56.2% specificity); low RPR → higher Karnofsky at 3 months
Matsubara et al., 2023 [15]	Retrospective Cohort	466, 9.3%	Blood	Eosinophils, PLTs	↓ PLTs and eosinophils ≥ 100/µL = independent recurrence risk factors
Puccio et al., 2023 [11]	Prospective Cohort	20, 42% (21% requiring reoperation)	Hematoma	CXCL9, MCP-1, G-CSF, IL-6, VEGF-A, IL-8, PDGF-AB/BB, PDGF-AA, MDC, IL-1α, IP-10, IL-5, RANTES, IL-10, IL-1β, IL-1 Ra, M-CSF	↑ IL-5 → ↑ GOS-E RANTES < 1649.24 pg/mL → ↑ recurrence

Abbreviations. ABO: ABO blood group system, APTT: activated partial thromboplastin clotting time, bFGF: basic fibroblast growth factor, β-TP: beta-trace protein, BNP-1: brain natriuretic peptide-1, BUN: blood urea nitrogen, CBC: complete blood count, CCL: C-C Motif Chemokine Ligand, CRP: C-reactive protein, CXCL: C-X-C Motif Chemokine Ligand, ECL: electrochemiluminescence, ELISA: enzyme-linked immunosorbent assay, EPCs: endothelial progenitor cells, ESR: erythrocyte sedimentation rate, FDP: fibrinogen degradation products, FXIII: factor XIII, GM-CSF: granulocyte-macrophage colony-stimulating factor, GOS-E: Glasgow outcome scale extended, Hb: hemoglobin, HDL: high-density lipoprotein, HGF: hepatocyte growth factor, IFN-g: interferon gamma, IL-: interleukin-, IL-2R: interleukin-2 receptor, IL-1RA: interleukin-1 receptor antagonist protein, INR: international normalized ratio, IP-10: interferon-γ-inducible protein 10, LEIN: latex-enhanced immune nephelometry, LDL: low-density lipoprotein, MCP-1: monocyte chemoattractant protein-1, M-CSF: macrophage colony-stimulating factor, MDC: macrophage-derived chemokine, NLR: neutrophil-to-lymphocyte ratio, NPR: negative predictive value, NR: not reported, PDGF-AA: platelet-derived growth factor-AA, PDGF-AB/BB: platelet-derived growth factor-AB/-BB, PIC: a2-plasmin inhibitor–plasmin complex, PLR: platelet–lymphocyte ratio, PLTs: platelets, PPV: positive predictive value, PT: prothrombin time, RANTES: regulated upon activation, normal T cell expressed and presumably secreted, RBCs: red blood cells, RDW: red blood cell distribution width, RPR: red blood cell distribution width to platelet count ratio, TG: triglycerides, TNF-a: tumor necrosis factor alpha, TPA: tissue plasminogen activator, TT: thrombin time, U/CR: urea-to-creatinine ratio, VEGF: vascular endothelial growth factor, WBCs: white blood cells, **↓**: reduced (levels/counts/events of), **↑**: increased (levels/counts/events of), → associated with.

## 4. Discussion

### 4.1. Biomarker Study Characteristics/Designs and Time Trends

Twenty-three studies published between 2003 and 2023 on a total of 3749 patients were identified in the present review. The median postoperative recurrence rate of 17.5% falls well within the range of previous estimates (5 to 30%) [2]. The notable limitations of the incorporated studies were their retrospective cohort/single-center designs (*n* =11 studies) and/or small cohort sizes (<100 patients, *n* = 12 studies).

A noteworthy trend is a shift in research focus towards circulating biomarkers on larger patient cohorts during the last four years (2020–2023: circulation focus in 12 of 13 studies, median patient size: 256 patients) compared with previous studies (2003–2018: circulation focus in 3 of 10 studies, median patient size: 63). While hematoma biomarkers allow for the analysis of local pathophysiological processes [3], the distinct advantages of assessing circulating prognostic biomarkers are the ease of sampling and that they offer the opportunity of tailoring patient treatment according to risk stratification preoperatively. In this context, current guidelines on cSDH treatment incorporate adjuvant drug therapies such as cholesterol-lowering statins and anti-inflammatory corticosteroids that are designed to improve patient neurological function and/or reduce recurrence [2,31]. From an analytical perspective, the protein analysis of biomarkers with significant findings ranged from single enzyme-linked immunosorbent [6,21,23], nephelometric [9] and electrochemiluminescent [28] assays to multiplex immunoassays [10,11,22].

### 4.2. Biomarkers in Recurrence Prediction

Numerous studies on biomarkers associated with inflammatory processes were identified. Enhanced levels of cytokine biomarkers in hematoma fluid have been highlighted as evidence for a robustly elevated local inflammatory index, as well as for the involvement of inflammatory processes in recurrence. The replication of findings was evident for IL-6 [6,21], with less consistent findings regarding the direction of change (increased or decreased levels) in recurrent cSDH for IL-8 and RANTES [10,11,21]. The notion of increased local anti-inflammatory activity (reflected by the levels of a panel of cytokine biomarkers) in association with reduced cSDH recurrence rates was introduced by the studies of a single group [10,22].

More recent studies assessing circulating cellular population ratios as markers of inflammation support the notion of a systemic inflammatory response in cSDH and its association with recurrence [18,19]. Thus, while Guresir et al. [18] proposed preoperative RPR as a novel independent predictor of cSDH recurrence, De Oliveira et al. [19] highlighted similar value for the postoperative NLR ratio. Of note, evidence for a systemic inflammatory response triggered by cSDH was previously obtained by studies comparing levels of cell inflammatory markers in patients and healthy controls [32,33].

Aside from the aforementioned cellular biomarkers, circulating eosinophil counts were indicated as independent predictors of recurrence by two studies, albeit with contradictory findings. Thus, Chen et al. [16] indicated that lower peripheral eosinophil counts are independently associated with recurrence, while Matsubara et al. [15] reported the opposite. The findings of Chen et al. appear consistent with evidence suggesting a protective role of hematoma-membrane-infiltrating eosinophils against recurrence in a previous histological study [34].

A substantial body of evidence supports a role for local coagulation and fibrinolysis in cSDH development [35,36,37,38,39], including evidence for excessive activation of the former during the early stages of development, succeeded by hyperfibrinolysis [40]. In relation to cSDH recurrence, Katano et al. [23] found higher levels of ΤPA, a major fibrinolytic mediator, in the hematoma fluid of patients that experienced recurrences, while Wang et al. [25] indicated lower levels of circulating fibrinogen in their recurrent cases. Circulating FDP (but not D-dimer) levels on patient admission as an independent predictor for recurrence was indicated by Hori et al. [27]. The authors of this study hypothesized that the systemic elevation of FDP induces a systemic inflammatory response, which in turn influences expansion of the hematoma and can lead to its recurrence [27]. The potential of coagulation cascade mediators as peripheral biomarkers in the prediction of cSDH recurrence is further supported by studies reporting preoperative thrombocytopenia or increased PT as independent risk factors for cSDH recurrence [15,26,41] and of lower circulating factor XIII in recurrent cases [24].

The formation of immature, leaky vessels in the outer membrane of cSDH upon which damaging forces of inflammation and of abnormal cerebral pulsations are exerted has been postulated as a driving mechanism for cSDH expansion, as well as a risk factor for its recurrence [3,20]. A study indicating significantly lower postoperative levels of peripheral EPCs in patients that experienced cSDH recurrences suggested that this decrease may lead to a reduced capacity for endothelium repair, thus increasing the risk of cSDH recurrence [20]. Following a similar logic, an attenuated protective role on vascular integrity and endothelial function due to decreased peripheral HDL levels was postulated by a second study indicating lower HDL levels as an independent predictor of recurrence [29].

Three additional studies indicated the potential value of peripheral biomarkers as independent predictors of recurrence. The dehydration status of cSDH patients on admission, estimated using the blood U/Cr ratio, was reported as an independent predictor of cSDH recurrence by Mainka et al., 2022 [30]. The authors proposed that the impact of dehydration is due to a reduction in brain volume and/or instigation of inflammatory processes that predispose patients to the occurrence/recurrence of cSDH [30]. Wang et al. investigated BUN levels in cSDH patients based on previous findings indicating the prognostic significance of BUN in neurological disorders such as ischemic stroke [25]. Their findings indicated that an elevated postoperative level of BUN is an independent risk factor for recurrence via mechanisms that remain to be established [25]. Blood type A was also highlighted as an independent risk factor for cSDH recurrence by Hirai et al. [14]. Nevertheless, it is worth pointing out that a second study on another Asian cohort, reporting significantly lower recurrence rates, did not find an association between ABO types and cSDH recurrence [41]. Larger prospective cohort studies that may include additional population samples are warranted to establish the impact of ABO blood types on cSDH recurrence.

### 4.3. Biomarkers in Neurological/Functional Outcome Prognosis

A smaller number of studies assessed biomarkers in relation to cSDH patient neurological/functional outcome. The pilot study of Puccio et al. indicated strong correlations between higher hematoma IL-5 levels and more favorable GOS-E scores [11]. Interestingly, higher hematoma IL-5 levels were associated with lower rates of cSDH recurrence in two other studies included in the present review [10,22]. Consistent with the notion of a significant association between a lower preoperative peripheral inflammatory index and a favorable functional outcome are the findings of Guresir et al. [18], indicating significantly higher Karnofsky scores 3 months postoperatively in the low compared to the high RPR patient group. Moreover, Idowu et al. [17] indicated significant associations between a low admission PLR, which represents another inflammatory marker, and poor outcome at 3 months using the GOS and LABDES. Notably, in the same study, inflammatory markers, such as c-reactive protein (CRP) and the NLR, did not display associations with the outcome [17].

Lastly, on the basis of previous evidence for significant associations between admission circulating BNP-1 levels and functional outcome in neurological disease, including traumatic brain injury and stroke, Chihi et al. [28] investigated circulating peptide levels in cSDH. Their findings indicated that preoperative BNP-1 is an independent predictor of functional outcome at 5–6 months postoperatively [28].

## 5. Conclusions

In the present systematic scoping review, we mapped and analyzed the pertinent literature on biofluid biomarkers showing promise in the prediction of cSDH recurrence and for the prognosis of neurological/functional patient outcomes. Twenty-three studies spanning three decades, analyzing a diverse range of biomarkers in hematoma fluid and/or the circulation, were included. Immune cell populations and inflammatory/anti-inflammatory cytokines comprised the most studied category of biomarkers displaying significant findings. A notable time trend in biomarker studies was a recent shift in research focus towards the analysis of circulating biomarkers on larger patient cohorts. Some of the studies on peripheral biomarkers highlighted the notion that aside from local inflammatory processes, cSDH triggers a systemic inflammatory response, and biomarkers associated with the latter are of prognostic value. Several biomarkers were indicated as independent predictors of cSDH recurrence and/or functional outcome, including circulating FDP, BNP-1 and HDL as well as BUN and blood NLR and RPR ratios. In recent years, studies on cSDH prognostic biomarkers have gained momentum. Nevertheless, additional multicenter prospective studies are warranted to confirm and extend their findings. The identification of prognostic biofluid biomarkers in cSDH is an active field of research that may provide future tools guiding clinical decisions and allowing the design of treatments based on risk stratification.

## Data Availability

Not applicable.

## Appendix A

The search strategy comprised the following: ((“chronic subdural haematoma”[All Fields] OR “hematoma, subdural, chronic”[MeSH Terms] OR (“hematoma”[All Fields] AND “subdural”[All Fields] AND “chronic”[All Fields]) OR “chronic subdural hematoma”[All Fields] OR (“chronic”[All Fields] AND “subdural”[All Fields] AND “hematoma”[All Fields])) AND (“biomarkers”[All Fields] OR “biomarkers”[MeSH Terms] OR “biomarkers”[All Fields] OR “biomarker”[All Fields])), ((“chronic subdural haematoma”[All Fields] OR “hematoma, subdural, chronic”[MeSH Terms] OR (“hematoma”[All Fields] AND “subdural”[All Fields] AND “chronic”[All Fields]) OR “chronic subdural hematoma”[All Fields] OR (“chronic”[All Fields] AND “subdural”[All Fields] AND “hematoma”[All Fields])) AND (“recurrance”[All Fields] OR “recurrence”[MeSH Terms] OR “recurrence”[All Fields] OR “recurrences”[All Fields] OR “recurrencies”[All Fields] OR “recurrency”[All Fields] OR “recurrent”[All Fields] OR “recurrently”[All Fields] OR “recurrents”[All Fields])), ((“chronic subdural haematoma”[All Fields] OR “hematoma, subdural, chronic”[MeSH Terms] OR (“hematoma”[All Fields] AND “subdural”[All Fields] AND “chronic”[All Fields]) OR “chronic subdural hematoma”[All Fields] OR (“chronic”[All Fields] AND “subdural”[All Fields] AND “hematoma”[All Fields])) AND (“prognosis”[MeSH Terms] OR “prognosis”[All Fields] OR “prognoses”[All Fields])).
